# Surgical Skills Olympiad: A 4-Year Experience in a General Surgery Residency Program

**DOI:** 10.1055/s-0041-1733991

**Published:** 2021-08-26

**Authors:** Kurun P. S. Oberoi, Akia D. Caine, Jacob Schwartzman, David H. Livingston, Aziz M. Merchant, Anastasia Kunac

**Affiliations:** 1 Division of General/Minimally Invasive Surgery, Department of Surgery, Rutgers New Jersey Medical School, Newark, New Jersey; 2 Division of Trauma and Surgical Critical Care, Department of Surgery, Rutgers New Jersey Medical School, Newark, New Jersey

**Keywords:** surgical simulation, surgical Olympiad, skills training, residency skills, residency training, surgical education

## Abstract

**Background** The acquisition of operative skills is the critical defining component of general surgery training. Performing simulated tasks has been shown to increase a resident's technical skills. As such, we devised the Surgical Skills Olympiad, an annual simulation-based skills competition. We examined our 4-year experience with the Olympiad at a large academic general surgery residency program.

**Objective** This study aimed to use competition to motivate trainees to increase the time they spent practicing basic surgical skills, resulting in improved performance over time.

**Methods** Teams were formed from members of each postgraduate year (PGY) class. Competition tasks were level specific: knot tying for PGY-1, basic laparoscopy for PGY-2, handsewn bowel anastomosis for PGY-3, vascular anastomosis for PGY-4, and advanced laparoscopy for PGY-5. Task scores over a 4-year period (2014–2017) were analyzed and a survey of participating teaching faculty was conducted.

**Results** Ten faculty members responded to the survey, for a response rate of 63%. A total of 50% respondents felt that the caliber of surgical skills increased since the Olympiad was implemented. Ninety percent agreed that the Olympiad was beneficial for residents to assess their skills against their peers. Over 4 years, there was an improvement in scores for suturing task, advanced laparoscopy, and bowel anastomosis ( *p* < 0.05 for all three).

**Conclusion** A residency-wide surgical skills competition can improve resident performance in technical tasks and promote faculty engagement in resident skills training.

Technical skill acquisition is a critical component of general surgery training. Faculty and residents have expressed concerns regarding trainees' technical ability. 1 2 Many surgical fellowship program directors (PDs) believe that trainees do not have adequate operative skills. 1 Residents also have concerns about their skill level and ability to perform independently in the operating room (OR). 2

Simulation has been shown to promote resident skill acquisition, 3 4 leading the Accreditation Council for Graduate Medical Education (ACGME) to require all general surgery programs to implement a simulation and skills curriculum. 5 There is, however, tremendous variation in how curricula are implemented.

Due to the concern regarding trainee operative ability and the known benefit of simulation for skills development, faculty at our institution devised the Surgical Skills Olympiad, a simulation-based competition among residents in our program. We hypothesized that competition between residents would result in improved performance over time during the Olympiad sessions.

## Methods

The Surgical Skills Olympiad, introduced in 2014, was an annual, 1-day competition at our institution between teams composed of residents. Approximately 6 to 8 teams each year were assembled. Each team had at least one resident representing each postgraduate year (PGY-1 through PGY-5). Sometimes, teams would have multiple junior level residents; however, the maximum number of teams each year was based on the number of graduating chief residents, so that each team had one chief resident leader. Tasks were PGY level specific ( Table 1 ). Teams were chosen randomly and announced well before the event, allowing time for teams to develop a plan before the competition.

**Table 1:** Surgical skills Olympiad tasks

Postgraduate year	Surgical skills
PGY-1	Suturing and knot tying
PGY-2	Basic FLS
PGY-3	Handsewn bowel anastomosis
PGY-4	Vascular anastomosis
PGY-5	Advanced laparoscopic intracorporeal suturing

Abbreviations: FLS, fundamentals of laparoscopic surgery; PGY, postgraduate year.

Teams were allowed as much training time as they wanted between the time that the teams were announced prior to the Olympia, and up to the morning of the Olympiad, as would be for any competition. The amount of time spent was individual and varied for each team.

The PGY-1 suturing and knot tying task was conducted on a homemade suture board previously validated for simulation of skin suturing. 6 The PGY-2 task consisted of laparoscopic peg transfers and pattern cutting conducted on fundamentals of laparoscopic surgery (FLS) training boxes. The PGY-3 task was a handsewn bowel anastomosis performed using porcine intestine purchased from a local butcher. The PGY-4 vascular anastomosis task was executed with 1/4-inch Penrose's drains and 1/2-inch polytetrafluoroethylene (PTFE) graft material with double-armed 6–0 prolene. Finally, PGY-5 intracorporeal suturing was conducted using FLS training boxes and 1-inch Penrose's drains with a midline defect to be closed by the resident in running fashion with 2–0 silk.

Tasks were graded on completion time (in minutes) with time penalties for as follows: (1) nonsquare knots and failure to approximate suture lines for the PGY-1 and -5 tasks; (2) dropped pegs or cutting outside the marked pattern for the PGY-2 tasks; and (3) anastomotic leak under water testing for the PGY-3 and -4 tasks. Faculty in the Department of Surgery scored all tasks. Each task had two faculty performing the grading of the tasks. Each task had a separate scoring system that involved time to completion, accuracy or performance, and penalties for errors.

The winning team was determined by aggregate score depending on their rank order for task score (see above). Specifically, for each task, the top five teams earned 10, 8, 6, 4, and 2 points respectively, based on their position rank for the completion of the task. Any remaining teams below the top 5 teams (in terms of performance) for each task received zero points. These points were then added across all tasks to determine the aggregate score. Teams who perform well across several PGY levels rather than in a single task were rewarded.

Task scores of Olympiad sessions from 2014 to 2017 were analyzed using nonparametric Kruskal–Wallis tests because of the low number of scores and skewed data. Statistical analysis was performed using the R statistical package. Summary statistics of task scores were calculated for each PGY level for comparable tasks. Due to changes in task configuration from year-to-year, PGY-1 knot tying and suturing scores were only compared in 2014, 2015, and 2017. The PGY-3 bowel anastomosis and advanced laparoscopic scores were only compared in 2015, 2016, and 2017 for the same reason. The vascular anastomosis (PGY-4) and basic FLS (PGY-2) tasks could not be compared due to differences in scoring between different years.

Faculty who participated in any of the four Olympiads were asked to complete an online survey following the 2017 competition. The surveys were completed on the day of the Olympiad by the faculty before they left the competition. Survey items assessed faculty perceptions of the Olympiad, both its ability to help residents evaluate their technical skills and improve those skills over time. Results were analyzed using simple summary statistics.

## Results

### Task Scores

We observed an improvement in median scores ( Table 2 ) for the suturing task for the years 2014, 2015, and 2017 (18 [median] ± 6.0 (interquartile range [IQR]), 8.3 ± 2.7, and 7.3 ± 1.8, respectively, *p* < 0.05]. Similarly, we observed an improvement in median scores for the bowel anastomosis task for the years 2015, 2016, and 2017 (13 ± 7.3, 19 ± 2.3, and 18 ± 1.0, respectively, *p* < 0.05) and for the advanced laparoscopic tasks for the same years (11.3 ± 1.6, 18.5 ± 3.9, and 19.3 ± 2.0, respectively, *p* < 0.05). We observed no difference in scores for knot tying over the same 3 years as the suturing task.

**Table 2:** Overall task scores (time in minutes) per year with year-to-year comparisons

Suture (PGY-1)					
Year	*n*	Median score	IQR	Actual IQR	*p* -Value
2014	13	18.0	16–22	6.0	<0.05
2015	19	8.3	7.73–10.45	2.7	
2017	17	7.3	6.4–8.19	1.8	
Knot tying (PGY-1)					
2014	13	5.0	0–8.0	8.0	0.58
2015	19	4.0	0–9.5	9.5	
2017	17	6.0	4–8.0	4.0	
Bowel anastomosis (PGY-3)					
2015	10	13.0	10.5–17.75	7.3	<0.05
2016	12	19.0	17.75–20	2.3	
2017	9	18.0	18–19	1.0	
Advanced laparoscopic (PGY-5)					
2014	6	11.3	10.58–12.225	1.6	<0.05
2015	7	18.5	15.065–18.975	3.9	
2016	8	19.3	18.1375–20.1375	2.0	

Abbreviations: IQR, Interquartile range; PGY, postgraduate year.

### Survey Results

Ten of 16 faculty completed the survey, for a response rate of 63%. Responses are shown in Table 3 . Ninety percent of faculty participated in at least 2 consecutive years. Fifty percent of faculty participated in all 4 years. Ninety percent of faculty (1) enjoyed participating in the Surgical Olympiad, (2) felt that resident competition was helpful in skill development, and (3) felt that the benefits of the Surgical Olympiad to the residents were worth the time commitment for faculty.

**Table 3:** Survey questions and results

Question	Responses		
	Disagreed or strongly disagreed	Neutral	Agreed or strongly agreed
I enjoyed participating in the Surgical Olympiad	10%	0%	90%
Resident competition is a good way for residents to assess their skill level against their peers	0%	10%	90%
Benefits of the Surgical Olympiad are worth the time commitment for attending surgeons who participate	10%	0%	90%
	Decreased	Remained the same	Increased
Since the first year of implementation, the caliber of surgical skills demonstrated at Olympiad has	0%	50%	50%

## Discussion

Several studies describe using competition to promote skill acquisition. Morris et al and Jiang et al reported improved medical student suturing technique following the introduction of a suturing skills competition. 7 8 El-Beheiry et al reported on the effect of competition in a laparoscopic training curriculum and found that residents had faster task times and increased voluntary practice compared with those who were not involved in the competition. 9 This has been replicated in other studies 10 and is consistent with our results which showed improved scores for suturing and advanced laparoscopic skills. Although we could not show an improvement in the bowel and vascular anastomosis tasks, studies have shown that practicing in these tasks in a noncompetitive 4 and competitive setting 11 results in an objective improvement in technique.

PDs acknowledge the importance of simulation for trainees but several obstacles to teaching and evaluating surgical skills have been identified, including lack of faculty time and support. 12 In our study, most faculty reported that they enjoyed participating in the Olympiad and it was worth their time commitment. Also encouraging is the fact that many faculty participated in at least 2 consecutive years. Implementing a skills competition is one way to increase faculty engagement in resident education.

Analysis of task-specific scores showed a significant improvement over time in PGY-1 suturing and PGY-5 advanced intracorporeal suturing. Faculty believed skills improved for all residents over the 4-year period, but there was no difference in scores for the PGY-2 through -4 residents. This could be due to a small sample size and variations in these tasks and scoring guidelines over time. Furthermore, for the PGY-3 bowel anastomosis task, the caliber of pig intestine varies year-to-year based on the size of animal slaughtered. Since task completion time is a consideration in final score, the score trends may not directly correlate to resident performance over time even though the scoring systems are comparable.

While our study provided insight into faculty perspectives regarding the value of a skills competition, we did not survey the residents. Anecdotally, many residents enjoy the competition, but it is unclear what effect it has on their motivation to practice and how often they practice. Other studies can provide us with some insight. McCreery et al found many residents felt that simulator practice improves intraoperative performance, and competition was a motivating factor to practice. 13 Verdaasdonk et al also found that motivation to win was a significant factor in driving residents to participate in a voluntary laparoscopic skills competition. 14 Interestingly, one study showed that implementing a statewide resident skills competition during the annual American College of Surgeons (ACS) chapter meetings was associated with a significant increase in resident attendance at these meetings, suggesting that competition could be used to increase resident engagement in other activities. 15

Another area to explore is using the competition to identify residents whose technical skills are lower than expected for their PGY level. This could promote early intervention for those having difficulty and allow for a remediation to occur.

One concern that we must not forget is potential for negative consequences from peer competition in an unhealthy form. Matsuo et al 16 conducted a cross-sectional survey with almost 60 residents to understand the causes of burnout, and resident peer competition during training was found to be independently associate with burnout.

## Limitations

This study has limitations. To add an element of surprise, some tasks were slightly changed from year-to-year. Because of this, we were only able to compare task scores for years in which the same task was performed. An additional limitation is our low survey response rate. Although not robust, it is on par with, and in some cases exceeds, the response rates seen for similar studies. 13 17 Another limitation is potential recall bias associated with a delayed survey. We could address this by performing a survey immediately after each competition. Finally, because tasks change for every PGY level, one cannot assess for skills retention over time. This could be addressed by having residents repeat their Olympiad task 6 months later, before it is changed.

## Conclusion

A residency-wide surgical skills competition can improve certain technical skills within the Olympiad framework and promote faculty engagement in the competition. Faculty find this time during the resident competition to be worthwhile.
